# Dual-Energy CT in Oncologic Imaging

**DOI:** 10.3390/tomography10030024

**Published:** 2024-02-23

**Authors:** Giovanni Foti, Giorgio Ascenti, Andrea Agostini, Chiara Longo, Fabio Lombardo, Alessandro Inno, Alessandra Modena, Stefania Gori

**Affiliations:** 1 Department of Radiology, IRCCS Ospedale Sacro Cuore Don Calabria, Via Don A. Sempreboni 5, 37024 Negrar, Italy; chiara.longo@sacrocuore.it (C.L.); fabio.lombardo@sacrocuore.it (F.L.); 2 Department of Biomedical Sciences and Morphological and Functional Imaging, University Hospital Messina, 98122 Messina, Italy; giorgio.ascenti@unime.it; 3 Department of Clinical Special and Dental Sciences, University Politecnica delle Marche, 60126 Ancona, Italy; 4 Department of Oncology, IRCCS Ospedale Sacro Cuore Don Calabria, Via Don A. Sempreboni 5, 37024 Negrar, Italy; alessandro.inno@sacrocuore.it (A.I.); alessandra.modena@sacrocuore.it (A.M.); stefania.gori@sacrocuore.it (S.G.)

**Keywords:** dual-energy CT, oncology, virtual non contrast, iodine map, monoenergetic

## Abstract

Dual-energy CT (DECT) is an innovative technology that is increasingly widespread in clinical practice. DECT allows for tissue characterization beyond that of conventional CT as imaging is performed using different energy spectra that can help differentiate tissues based on their specific attenuation properties at different X-ray energies. The most employed post-processing applications of DECT include virtual monoenergetic images (VMIs), iodine density maps, virtual non-contrast images (VNC), and virtual non-calcium (VNCa) for bone marrow edema (BME) detection. The diverse array of images obtained through DECT acquisitions offers numerous benefits, including enhanced lesion detection and characterization, precise determination of material composition, decreased iodine dose, and reduced artifacts. These versatile applications play an increasingly significant role in tumor assessment and oncologic imaging, encompassing the diagnosis of primary tumors, local and metastatic staging, post-therapy evaluation, and complication management. This article provides a comprehensive review of the principal applications and post-processing techniques of DECT, with a specific focus on its utility in managing oncologic patients.

## 1. Introduction

Dual-energy CT (DECT) has become an integral component of clinical practice, offering a wide array of applications across various medical domains, including musculoskeletal, vascular, cardiac, gastrointestinal, genitourinary, and neurological fields [1,2,3,4,5,6,7,8,9,10,11,12,13,14,15,16,17,18,19]. Notably, recent advancements have highlighted its significant advantages in the evaluation of oncology patients [20,21,22]. Key among these applications are virtual non-contrast (VNC) and iodine maps, which effectively highlight tissue vascularity [23,24,25,26,27,28,29,30,31,32,33,34] in both primary tumors and metastases [35,36,37,38,39,40,41,42,43]. Furthermore, enhanced visualization of vascular structures enables the optimized assessment of vascular involvement [44]. Virtual monoenergetic reconstructions further enhance visualization, particularly of small lesions, by increasing tissue contrast and reducing artifacts [45,46,47,48].

The radiologist faces an oncological patient with numerous management needs, including the identification of the primary tumor, establishing its relationships with neighboring structures, and objectively and repeatably evaluating morphology and density. This lays the groundwork for a primary diagnosis, for follow-up, and for a reliable reassessment after targeted therapies. Furthermore, in some patients, it will be necessary to make a differential diagnosis of any incidental findings. 

In comparison to previous studies in the literature, our paper focuses on a comprehensive and precise review and on a discussion of DECT applications, categorized by specific application, emphasizing their significant contributions to the management of oncology patients.

### Imaging Technique

There are various types of dual-energy CT scanners available, each with different imaging acquisition technologies based on the vendor [49]. The common factor among these systems involves having two sets of image datasets with different energy levels (keV) which, through dedicated software applications, allow for optimized CT diagnoses. Specifically, the scans can be acquired using different methods such as dual-source tubes, rapid switching of energy levels within the same tube (fast kV switching), sequential scans, or using panels characterized by different layers. 

Dual-source CT employs two X-ray sources and detector arrays positioned at different angles. This technology allows for the simultaneous acquisition of data at different energy levels, typically high and low kilovoltage (kV), enabling the production of dual-energy images. This approach enables the acquisition of images with higher iodine contrast-to-noise ratio, reduced beam-hardening artifacts, and the possibility of material-specific images [50,51]. In the rapid kV switching technique, a single X-ray tube rapidly switches between two energy levels to obtain dual-energy data. This method is implemented in some modern CT scanners, allowing for the acquisition of dual-energy information. The sequential scan approach involves acquiring images at different energy levels sequentially, rather than simultaneously. This method enables the generation of dual-energy information by capturing images at distinct energy levels, one after the other, during the scanning process. In a dual-layer detector, CT scanners are equipped with detectors with two layers that differentiate the energy levels of the incoming X-rays. This design enables the acquisition of dual-energy information in a single scan. 

Lastly, the most recently released technical innovation, photon-counting detector computed tomography (PCD-CT), offers greater capabilities in multienergy CT as well as spatial resolution, directly detecting and counting individual photons [50].

The applications described and the clinical cases shown in this review paper were acquired using different scanners employing different technologies as the underlying principles of the applications remain the same (for instance, for virtual non-contrast and iodine map acquisitions).

Theoretically, any phase of a CT scan can be acquired using the dual-energy mode. Generally, in clinical practice, dual-energy acquisitions focus on arterial scans and on venous scans, which are pivotal for CT-based oncology diagnosis [52,53]. Arterial images allow for the dedicated study of arteries and bleeding [54]. Furthermore, the arterial phase is typically necessary and ideal for identifying hypervascular tumors and metastases [55]. Among the most highly vascularized tumors, better identified in the arterial phase, are hepatocellular tumors, which typically display intense wash-in during the arterial phase [56]. Other tumors that are typically well-vascularized in the early stages of dynamic imaging include renal tumors, particularly clear cell tumors [55]. In the adrenal region, paragangliomas demonstrate typical arterial vascularity, as do the majority of endocrine tumors, both functional and non-functional. Among these, carcinoid tumors, aside from being highly vascularized, are often associated with hypervascular metastases, frequently localized in the hepatic area and in the mesenteric lymph nodes [55]. Many metastases, especially in the abdominal region, often demonstrate vascularity in the arterial phase, including melanoma metastases that can affect virtually any body district, particularly the superficial soft tissues. 

Further to this, the enhancement in tissue contrast achieved in dual-energy during the arterial phase can help better define the presence of hypovascular tumors, especially in glandular areas, where healthy glandular tissue stands out against the hypovascular tumor area. This is the case, for example, in pancreatic tumors, as well as in tumors originating from the bile ducts.

Conversely, venous images offer insight into peritumoral vascular involvement [57]. Also, the venous phase acquired in dual-energy imaging can theoretically benefit from the greater density difference between healthy tissues and those involved in the tumor process, especially in lesions that exhibit wash-out in venous phase. Among these, in addition to primary hepatocellular liver tumors, there are most hypovascularized liver metastases, which are among the most frequent in daily practice, affecting patients with colon, breast, and lung cancer. Non-contrast dual-energy acquisition could also be beneficial in improving the performance of applications focused on studying bone, liver, and calcifications, which may be associated with pancreatic or obstructive diseases [29,41]. These acquisitions are also the most commonly used and likely more reliable for opportunistic assessment of vertebral density, which can be utilized as an alternative to conventional bone densitometry for identifying osteoporosis. Osteoporosis, affecting a significant number of patients, particularly many post-menopausal women, represents a significant comorbidity in women with cancer, where the condition can certainly be exacerbated by frequently necessary ongoing therapies. Similarly, baseline DECT acquisitions can be employed to assess muscle trophism, which could represent another cornerstone in the management of oncology patients, potentially serving as an important indicator of overall health deterioration, thereby acquiring potential prognostic and management value.

## 2. Virtual Non-Contrast (VNC)

The virtual non-contrast (VNC) application is widely utilized in clinical practice, particularly in oncology patients. Among its major advantages, this application allows for the possibility to not acquire the baseline scan, potentially reducing radiation dose, as it is a critical factor for oncology patients undergoing multiple serial CT scans [24]. Along with providing high-quality images, VNC datasets enable reliable and dedicated densitometric measurements, which have practical clinical utility [58].

### Applications

**Figure 1:**
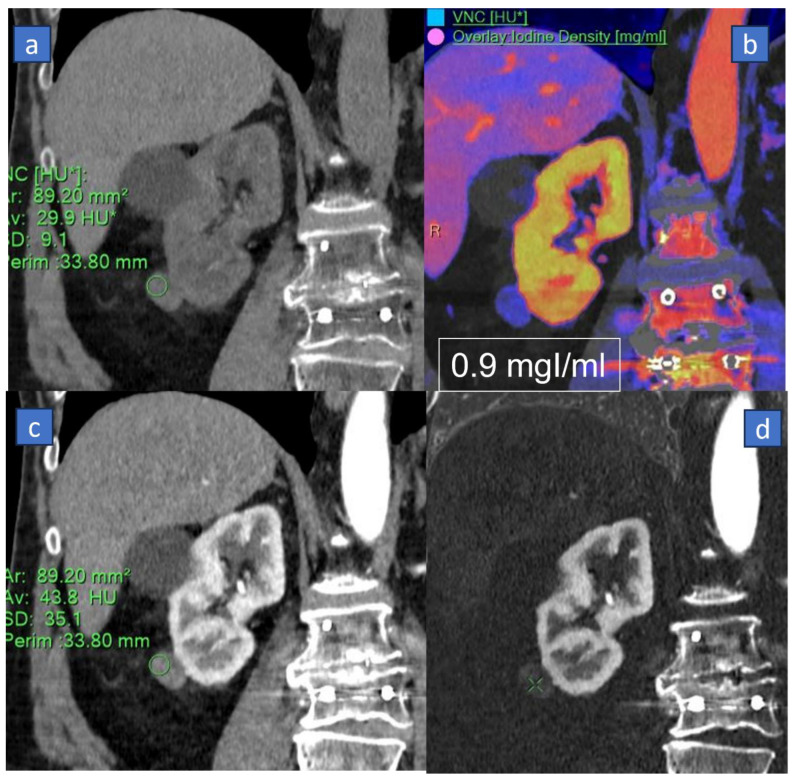
Sixty-eight-year-old woman suffering from incidental surgically proven papillary carcinoma of the right kidney. On the coronal reconstructed conventional baseline CT, (**a**) a small exophytic inferior polar nodule of the right kidney can be recognized (circular region of interest) with solid density (29.9 HU). On the corresponding iodine density superimposed on the virtual non-contrast image on the coronal plane, (**b**) the lesion shows a clear iodine uptake (0.9 mgI/mL), with different behavior compared to the simplex cyst located on the mid third of the same kidney. On the corresponding coronal contrast enhanced CT image, (**c**) the lesion demonstrates equivocal enhancement (43.8 HU), and the density could be affected by artifacts due to metal spine fixation. The qualitative assessment of the corresponding coronal subtracted image (**d**) shows only a mild equivocal enhancement.

One of the most important and reliable fields of application of VNC is renal imaging. In this scenario, the VNC application aids in distinguishing between benign renal cysts and solid renal masses without the need for a separate non-contrast acquisition (Figure 1). Indeed, renal cysts represent a common incidental finding in oncologic patients, and many cysts may show septations, internal nodules, or hyperdense content due to intra cystic hemorrhage or increased protein content. Also, new renal cysts may represent a relatively common finding in follow-up studies. VNC can help assess the internal content of the renal cysts, differentiating between simple cysts (which are fluid-filled and typically benign) and complex cysts or solid lesions (which may indicate malignancy or other concerning features) [59]. In clinical practice, benign renal cysts usually exhibit attenuation close to that of water on non-contrast images, appearing hypodense or with low attenuation values. In contrast, complex cysts or solid lesions show varying attenuation values or enhancement patterns on contrast-enhanced images, indicating the presence of internal structures, septations, calcifications, or solid components. This differentiation is crucial for appropriate patient management, determining further imaging or follow-up studies and guiding treatment decisions. In oncologic patients, this approach is of fundamental importance in cases of incidental cystic lesions to avoid repetition of the true non-contrast scan, reducing radiation burden and patient’s discomfort.

In the paper by Çamlıdağ et al., using histopathological diagnosis as a reference for diagnosis, the authors assessed the value of dual-energy CT in distinguishing benign versus malignant renal lesions, with a significant difference being found between the iodine content of clear cell and non-clear cell (papillary + chromophobe) RCC (*p* < 0.001), and in distinguishing aggressive versus indolent lesions [60]. Moreover, the authors proposed a cut-off as concerns iodine content in differentiating clear cell from non-clear cell RCC (3.2 mg/mL), and a significant difference was found between the attenuation values of true and virtually unenhanced images (*p* = 0.007) [60]. 

Another application is the assessment of incidental adrenal findings [26,27]. Adrenal metastases are relatively common in oncologic patients, especially with lung, breast, colon, and kidney cancers. The primary step in the differential diagnosis involves evaluating the baseline density of the nodule. Densitometric values below 10 HU usually indicate an adenoma [61], while higher values raise suspicion of metastasis. This differential diagnosis is not only prognostically relevant for the patient but also substantially impacts management decisions.

In this scenario, even in patients without a true baseline acquisition, densitometric measurements can be utilized on VNC reconstructions [26,27]. While there is typically a discrepancy of about 5–6 HU between densitometric values between the true pre-contrast scan and the virtual one (depending on scanner type and imaging parameters), these values can still be employed in daily practice. Specifically, it is advisable to maintain a safety margin, often around 10 HU. For instance, an adrenal lesion with internal VNC density measuring around 0 HU can confidently be considered an adenoma, whereas any lesion with density values higher than 20 HU should be considered as metastases, and wash-out curves should be acquired [62]. Through VNC imaging, DECT can also efficiently detect the hyperdensity characteristic of adrenal hemorrhage, where the attenuation value is greater than other masses (50–70 HU), and usually, no appreciable contrast enhancement is noted [63]. 

**Figure 2:**
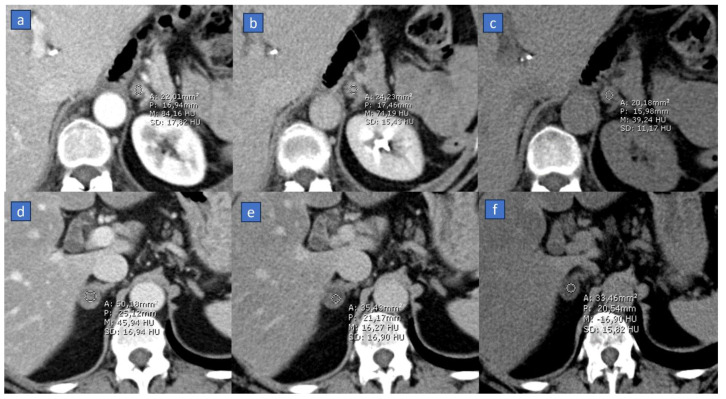
The role of the virtual non-contrast (VNC) application in oncologic patients with incidental adrenal lesions. In the first case, a 45-year-old male suffering from lung cancer with a small solid nodule of the left adrenal gland diagnosed as a metastasis. On dynamic axial scan (**a**), the lesion shows discrete enhancement, with slow wash-out in the 15-min scan (**b**). On the corresponding VNC scan (**c**), the lesion shows density >10 HU (39 HU), which is consistent with the diagnosis of a metastasis. In the second case, an adrenal adenoma in a 39-year-old woman with breast cancer shows mild enhancement during the venous phase (**d**), fast wash-out (**e**), and above all, a clear adipose density (−16 HU) on the corresponding VNC scan (**f**).

Moreover, in adrenal diagnostics, the baseline scan aids in calculating absolute and relative washout [61]; by employing a similar safety margin, appropriate washout curves can be calculated even in the absence of a true baseline scan (Figure 2).

In vascular imaging, particularly in computed tomography angiography, VNC allows for the assessment of vascular calcifications without the need for a separate non-contrast scan [64]. It distinguishes between areas of calcification and contrast-enhanced blood vessels, providing insights into the extent and distribution of vascular calcifications. Also, VNC allows for the differentiation of iodine contrast from pathology, aiding in the identification of vascular pathologies such as aneurysms, dissections, or thrombosis [65]. 

Also, VNC can be employed for the evaluation of bleeding or hemorrhage in combination with iodine maps. These conditions may represent threating complications in oncologic patients both during the diagnostic pre-treatment work-up and during follow-up studies. These conditions may represent complications after surgery or following chemo- or radiotherapy.

The VNC application in gastrointestinal bleeding serves as a valuable tool in diagnostic imaging. This application allows for the visualization and assessment of blood in the GI tract without the need for a conventional non-contrast CT scan. It aids in distinguishing areas of high-density blood from surrounding tissues or fluids within the GI tract, providing critical information to localize the source and extent of bleeding. VNC, especially if combined with iodine maps, can enhance the detection of subtle active bleeding [9]. In the paper by Sun et al., lower noise and higher SNR were found on VNC images than on true-non-contrast images (*p* < 0.05) with similar image quality (*p* > 0.05) [33]. By using DECT, the AUC in depicting active bleeding source was 0.95 for portal–venous phase with dual-energy mode and post-processing VNC datasets and iodine map, representing an accurate screening method with a lower radiation dose [33].

In recent years, a limitation in the utilization of VNC imaging has been identified in the significantly higher mean attenuation and higher noise levels compared to true non contrast phases, for example, in the arterial-phase CT angiography [66] and in the calcifications subtractions [67,68]. However, advancements in the latest generation of DECT have shown improvements in addressing these issues, as evidenced by several studies [69,70,71,72]. 

## 3. Iodine MAP

Another important DECT application in oncologic imaging involves the use of iodine maps, which are based on the principle of the sudden increase in absorption of X-ray photons by iodine at a specific energy (k-edge) [73]. Iodine is a fundamental component of contrast agents used in CT in clinical practice, commonly administered to highlight vascular structures and lesions that might otherwise be indistinguishable from surrounding healthy tissue. Several studies have demonstrated that iodine maps in DECT offer high sensitivity and specificity in visualizing the concentrations and distribution of iodine in tissues, effectively highlighting their vascularity [35,36,39,41,43,53,57].

### 3.1. Applications

**Figure 3:**
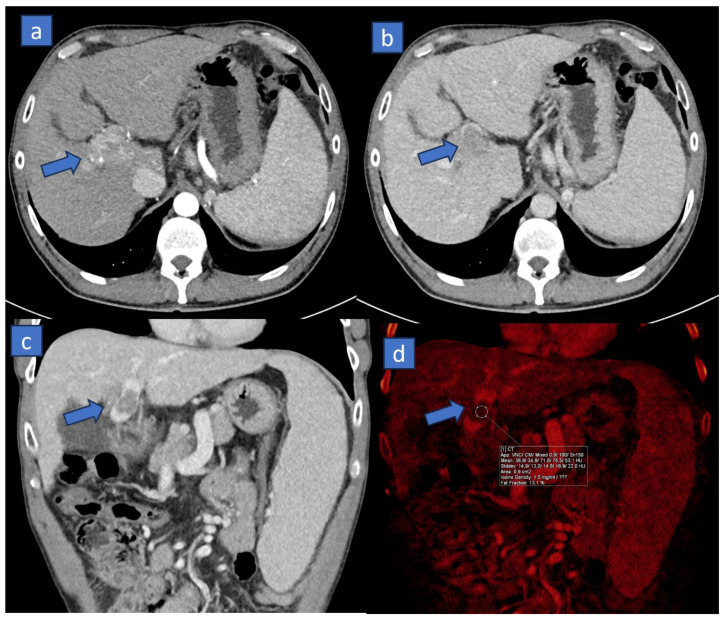
Sixty-five-year-old man suffering from liver cirrhosis with solid neoplastic thrombus of the portal vein. On the axial CT scan acquired during arterial phase, (**a**) a hypervascular liver tumor is depicted on hilum region, consistent with the diagnosis of hepatocellular carcinoma (arrow). On the corresponding venous-phase scan, (**b**) the lesion shows the typical wash-out pattern (arrow). On the coronal CT reconstruction, (**c**) a hypodense filling defect is recognized inside the portal vein (arrow). On the corresponding DECT reconstruction, (**d**) the assessment of DECT numbers (mean density: 36.9 HU; iodine density: 1.5 mg/mL) calculated from iodine map shows a clear enhancement of the solid neoplastic thrombus (arrow).

The main applications of iodine maps in DECT include the diagnosis and monitoring of neoplasms; in particular, the use of iodine maps allows for the better delineation of lesions that might otherwise be poorly visible due to their size or their being embedded in the context of heterogeneous tissue, such as that of a cirrhotic liver [6]. The extent of hypervascular patterns demonstrated by HCC can vary significantly and depends on several factors, including the location and size of the lesion, the timing of each individual scan post-contrast administration, and of course, the extent of both the arterial supply to the lesion and the venous supply to the liver. The lesion will be more visible the greater the density difference compared to the surrounding glandular tissue. In clinical practice, hypervascularity is undoubtedly among the most important criteria for diagnosing HCC. The diagnosis holds fundamental management and prognostic value. Increasing the density difference between hypervascular lesions and the surrounding liver can be facilitated by various applications favored in dual-energy imaging, primarily iodine maps, as well as monoenergetic imaging, where keV values can be optimized retrospectively to enhance the visualization of highly vascularized structures. Also, it should be emphasized that in abdominal imaging, particularly in liver assessments, acquiring dual-energy CT not only in the arterial phase, to better visualize the increased vascularity pattern of primary tumors, but also in the venous phase, to better evaluate the presence of lesion wash-out, could be beneficial. Moreover, during this phase, dual-energy acquisitions can aid in visualizing and differentiating thrombi (Figure 3). Neoplastic thrombi, in fact, will demonstrate a solid density and a clear uptake of iodine, typical of vital and vascularized tissues. In this scenario, DECT has been proposed for grading hepatic fibrosis via the extracellular volume fraction from iodine mapping in spectral liver CT [74]. 

In a recent study by Li et al., the diagnostic performance of dual-phase contrast-enhanced multiparametric DECT was evaluated in combination with deep learning radiomics in diagnosing macrotrabecular-massive subtype hepatocellular carcinoma (HCC) using histopathologic findings after surgery as a reference standard. They found that DECT can accurately predict the said subtype, with AUC ranging between 0.87 and 0.91 [75].

Some preliminary studies also suggest the possibility of characterizing lesion nature based on quantitative measurements from iodine maps. These maps can be utilized post-chemoembolization, thermal ablation, or cryotherapy to assess the perfusion of the treated area and response to therapy [76]. In particular, color-coded images are also used to effectively discriminate contrast-enhanced lesions from compact iodized oil accumulations in lesions treated with TACE, aiming to identify viable lesions around the hepatocellular carcinoma [77].

In patients with suspected pancreatic cancer, iodine maps might aid in distinguishing between pancreatic carcinoma and mass-forming pancreatitis. In fact, DECT can help in increasing the tumor conspicuity and margin sharpness and can help in the reproducibility of primary tumor measurements [78]. Also, due to typical hypervascular pattern shown by the majority of pancreatic endocrine tumors and by their metastases, iodine maps may represent a powerful tool for local staging and for the evaluation of distant metastases [79].

**Figure 4:**
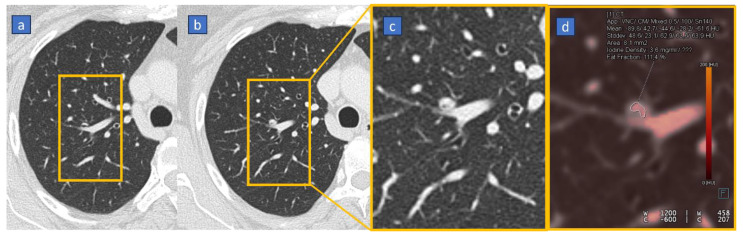
Fifty-one-year-old woman with surgically proven endobronchial tumor. On the baseline high resolution CT scan, (**a**) there is no evidence of tumor. During the one year follow up study, (**b**) there is evidence of a tiny endobronchial filling defect. On the corresponding contrast enhanced cropped axial image (corresponding to yellow box), (**c**) it is not possible to distinguish between a mucoid plug and a solid tumor. On the corresponding contrast enhanced DECT iodine map image, (**d**) it is possible to demonstrate a subtle iodine uptake, confirmed on the quantitative assessment achieved by placing a free hand ROI on the tumor.

Additionally, iodine maps can be employed for characterizing lung nodules [80]. In the study by Lennartz et al., 183 cancer patients who underwent contrast-enhanced venous phase of the chest were included. Volumetric HU attenuation and iodine concentration were used for differentiation of lung nodules. Monoparametric lung nodule differentiation based on either feature alone (i.e., attenuation or iodine concentration) was poor (AUC = 0.65), although the most powerful iodine map-derived feature slightly, yet insignificantly, increased classification accuracy compared to classification based on conventional image features only [80]. Iodine uptake can be key to the characterization of small endobronchial filling defects. In fact, endoluminal plugs, although hyperdense, do not show any significant enhancement or iodine uptake. Conversely, even a small endobronchial tumor could be characterized as a small solid lesion because of measurable iodine content (Figure 4).

Further to this, DECT has been employed for the identification and characterization of colon tumors [81]. In particular, in the paper by Özdeniz et al., the dual-energy CT characteristics of colon and rectal cancer allowed for their differentiation from stool. Notably, all colorectal tumors in said study showed homogeneous patterns on an iodine map. In particular, the density of stools was significantly lower than tumors in both iodine map and VNC images (*p* < 0.001).

In brain tumors evaluation, DECT can help in highlighting areas of recent hemorrhage, whereas in the head–neck region, DECT cab be utilized to differentiate between cartilaginous structures and neoplastic infiltration [82]. 

Furthermore, it has also been demonstrated that the concentration of iodine varies significantly among benign, inflamed, and neoplastic lymph nodes, with lower iodine uptake in metastatic than non-metastatic lymph-nodes [83].

**Figure 5:**
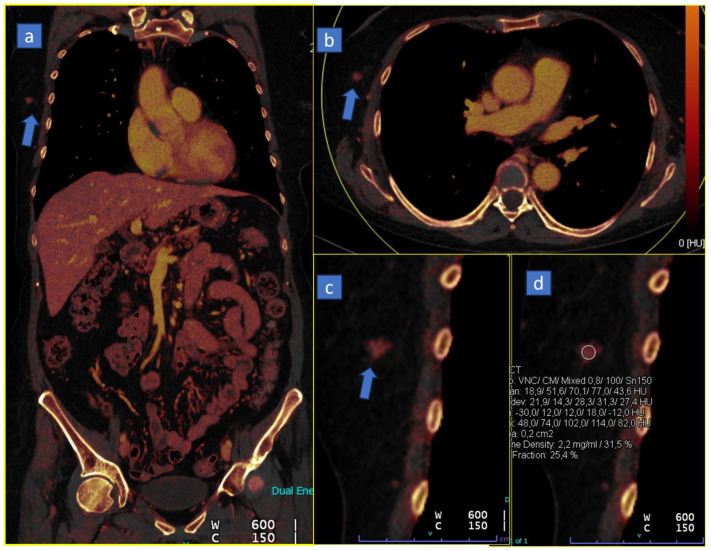
Seventy-one-year-old woman suffering from rectal cancer with incidental breast cancer on a DECT scan. On the coronal DECT scan acquired during venous phase, (**a**) a tiny hypervascular breast nodule is recognized (arrow). On the corresponding axial scan, (**b**) the lesion shows irregular margins (arrow). On the coronal CT cropped image, (**c**) the nodule shows clearly spiculated margins (arrow). By positioning an ROI (**d**), the assessment of DECT numbers calculated from an iodine map shows a clear enhancement of the solid tumor (ROI).

Iodine maps in imaging, particularly in CT scans using dual-energy technology, play a crucial role in the detection and characterization of incidental lesions. These maps allow radiologists to differentiate between different tissues and pathologies based on their iodine content, providing additional information beyond standard grayscale images. First, iodine maps contribute to detecting incidental lesions because of enhanced contrast enhancement, highlighting the iodine uptake within tissues. Incidental lesions that contain iodine can be more conspicuous on these maps compared to conventional images, aiding in their detection (Figure 5). Also, the presence of a measurable iodine uptake allows for improved lesion characterization. In the scenario of an incidental detected lesion, especially when the baseline is not available, iodine maps help differentiate between vascularized and non-vascularized lesions. An increased iodine uptake usually indicates increased vascularity, which might suggest malignancy or active pathological conditions. These images provide quantitative data on iodine concentration within tissues, enabling radiologists to analyze and measure the degree of iodine uptake in incidental lesions. These quantitative data assist in making a more precise diagnosis and characterization of the detected lesions, aiding in the differential diagnosis between benign and malignant lesions. Moreover, the increased contrast resolution allows us to better delineate tumor margins, which may help in the differential diagnosis or prognostic assessment. 

**Figure 6:**
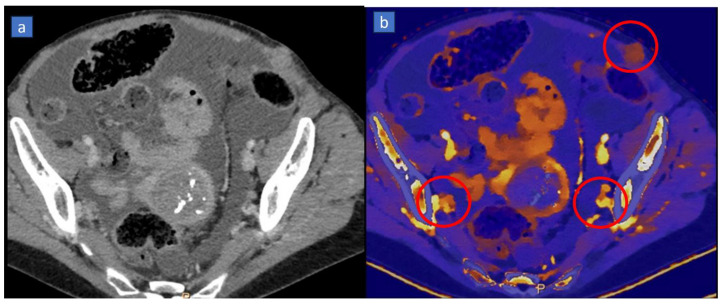
Staging CT in a 49-year-old patient with metastatic ovarian carcinoma. On an axial contrast-enhanced CT image, (**a**) the presence of ascites allows us to recognized multiple subtle peritoneal nodules. On the color-coded iodine maps, (**b**) metastatic peritoneal implants (red circles) can be clearly detected because of the avid iodine uptake.

Dual-energy CT (DECT) imaging is valuable in detecting distant metastases, especially in abdominal cavities (Figure 6). Peritoneal metastases refer to the spread of cancerous cells to the peritoneum, the membrane lining the abdominal cavity. DECT offers improved contrast resolution compared to conventional CT scans. It allows for better tissue characterization, enhancing the detection of peritoneal nodules or masses that might indicate metastases. Dual-energy CT provides material decomposition techniques that help differentiate between different tissues based on their material composition. This differentiation aids in distinguishing between normal abdominal structures and abnormal tissues indicative of metastases. This is particularly true when dealing with vascularized peritoneal implants, clearly visualized on iodine maps (Figure 6) because of their different blood supply pattern compared to surrounding healthy tissues.

### 3.2. Response to Therapy

In addition to aiding in diagnosis, iodine maps allow for the evaluation of the response to therapy [84,85,86]. Some lesions initially respond to therapy with a reduction in vascularity (and therefore iodine uptake) rather than a reduction in size [86], which is particularly true in post-chemotherapy evaluations with antiangiogenic drugs or tyrosine kinase inhibitors. In the study by Fervers et al. [84], therapy response of multiple myeloma after radiotherapy was monitored by using virtual non-calcium imaging, which yields potential for optimizing the lesion-specific radiation dose for local tumor control. In this study, decreasing attenuation indicates RT response, while above-threshold attenuation lesions precede local irradiation failure. Interestingly, this study only employed a non-enhanced DECT scan. In particular, VNCa CT was significantly superior for the identification of radiotherapy effects in lesions with higher calcium content (AUC of DECT 0.96 versus AUC 0.64 for conventional CT). Hellback et al. evaluated the potential role of DECT to visualize antiangiogenic treatment effects in patients with metastatic renal cell cancer (mRCC) treated with tyrosine kinase inhibitors (TKI) [86]. The authors enrolled 26 patients with mRCC, studied at baseline and follow-up with a single-phase abdominal contrast-enhanced DECT scan. VNC and iodine images were employed to evaluate 44 metastases. In particular, Hounsfield unit (HU) values and iodine density (ID), as well as iodine content (IC) in mg/mL of tissue, were compared to the venous-phase DECT density. Between baseline and follow up CT, the relative reduction measured in percent was significantly greater for iodine density than for standard CT density (49.8 ± 36.3% vs. 29.5 ± 20.8%, *p* < 0.005). IC was also significantly reduced under antiangiogenic treatment (*p* < 0.0001). For this reason, a sign of tumor response to antiangiogenic treatment is reduced tumor perfusion. DECT allows for a more sensitive detection of antiangiogenic treatment effects in vascularized metastases, as with those in metastatic clear cell carcinoma.

### 3.3. Organ Perfusion

Iodine mapping can be used to assess organ perfusion, for example, ischemic or inflamed intestinal walls, ovarian vascularization following torsion, and myocardial perfusion after a heart attack [87]. In a recent paper, lung perfusion in the context of pulmonary embolism was evaluated in a cohort of oncologic patients [88]. DECT was successfully employed for the visualization of thromboembolism and for the visualization of hypo perfused areas. In particular, by using a combination of lung analysis application and monoenergetic reconstruction, in the per-patient analysis, venous-phase images yielded a sensitivity and specificity of 90.0% and 100%, respectively, for both readers, in comparison to standard arterial-phase images [88]. This paper demonstrates that DECT applications can be used in oncologic patients for incidental diagnosis of thromboembolism in the lung, reducing the need for a dedicated additional pulmonary arterial scan.

## 4. Virtual Monoenergetic

The wide range of images generated from DECT acquisitions provide several advantages, such as improved lesion detection and characterization, superior determination of material composition, reduction in the dose of iodine, and more artifact reductions [89].

One of these applications is the energy-specific post-processing reconstruction of advanced virtual monoenergetic images (MEI+). Images created from a dual-energy CT dataset can simulate those acquired with a monochromatic beam set between 40 and 190 keV. In this context, the higher-energy beam (typically set to 140 kV or higher) is useful in the attenuation of beam hardening, while the lower-energy beam (80 kV or lower) provides superior soft tissue contrast and better contrast media conspicuity because the k-edge of iodine is 33.2 keV. 

### 4.1. Better Conspicuity of Lesions 

Creating a virtual monoenergetic beam offers the possibility of having a better conspicuity of vascularized lesions through the highlighting of the contrast material at low-voltage images secondary to the higher attenuation of iodine, which are best suited for the assessment at lower voltages as they have a better signal-to-noise ratio as compared to polychromatic images. This is particularly useful in the oncologic field, both in terms of the identification of lesions, characterization, follow-up, and response to therapy. These applications can be used in multiple fields to identify both primary lesions and for secondary lesions. 

Focusing on the liver, it is possible to increase the conspicuity of small secondary lesions (especially if less than 7 mm) at MEI+, maximizing the tumor-to-liver contrast and contrast-to-noise ratio at 40 keV without increase in the image noise of the remaining hepatic parenchyma [90].

**Figure 7:**
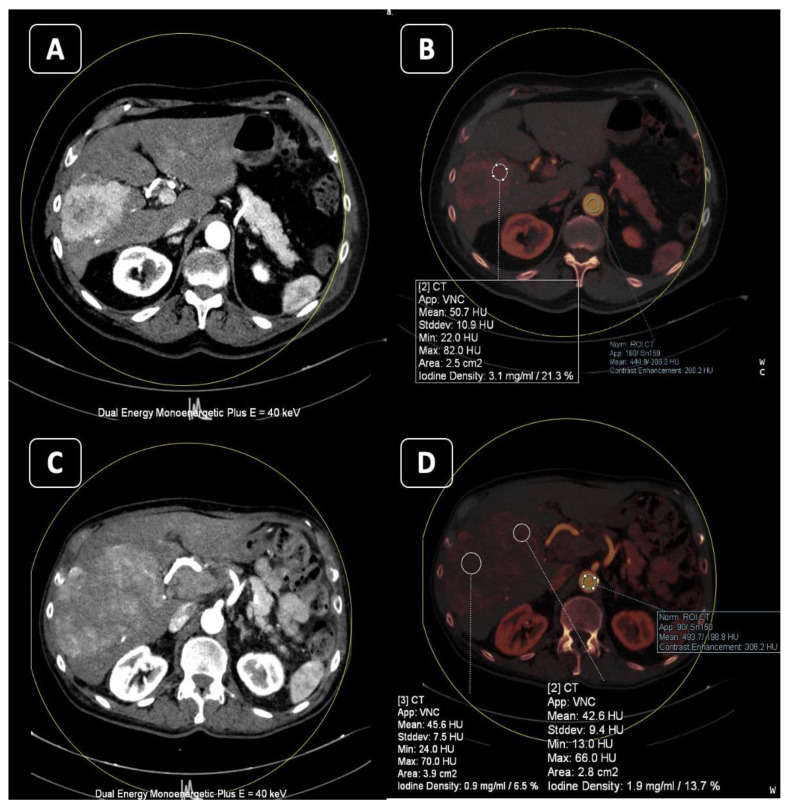
Eight-one-year-old male with hepatocellular carcinoma. On an axial reconstructed virtual monoenergetic image, (**A**) in arterial phase at 40 KeV, the tumor is clearly hypervascular with respect to spared parenchyma. On the corresponding axial DECT iodine map, (**B**) the lesions shows high iodine density (3.1 mg/mL). On the follow-up during treatment with Lenvatinib (**C**,**D**), although the lesion has increased in size, there is a significant reduction in the vascular pattern in the virtual monoenergetic reconstruction (**C**) and in iodine density on the iodine map (1.9 mg/mL) as a partial response to therapy.

A similar argument applies to primary hepatic lesions, primarily hepatocellular carcinoma (HCC), which is better identified thanks to enhancement in the arterial phase (Figure 7) and wash-out in the portal and delayed phases [91].

The low monoenergetic images also provide the highest contrast-to-noise ratio and signal-to-noise ratio for the detection of pancreatic ductal adenocarcinoma (PDAC). In fact, many studies have demonstrated that lesion conspicuity was significantly higher in monoenergetic images at 55 keV, with overall increased reader confidence at 70 keV [11,22,92,93]. Gupta et al. have found that the use of VMI with 50 keV and 70 keV images plays a very important role since it could be a problem-solver in cases of pancreatic adenocarcinoma [78].

For the study of typical hypervascularized or atypical hypoattenuating and isoattenuating insulinomas, the efficacy of MEI+ images in better optimizing contrast has also been demonstrated [94].

The MEI reconstructions proved to be helpful for differentiating simple cysts from cystic tumors both in renal and ovary tumors, given the increased conspicuity of the cyst wall and septa in low-keV images [95]. In particular, a study by Patel et al. found that sensitivity and specificity for the thresholds did not change significantly between low-energy and 70 keV virtual monoenergetic imaging, with the AUC at 40 keV being 0.96 and 0.98 at 70 keV [96]. 

In the gastrointestinal tract, DECT at low-keV monochromatic images can be used to better delineate duodenal adenocarcinoma extent or to improve visualization of early-stage gastric cancers [97].

For the detection of benign salivary gland tumors, DECT has been used in the identification of pleomorphic adenomas since they may not be appreciated on contrast-enhanced conventional CT because of the poor enhancement and low overall density of the parotid gland [98].

### 4.2. Less Contrast Material

Another advantage of post-reconstruction DECT at lower keV is its higher sensitivity for iodine that permits the reduction in the amount and concentration of intravenous contrast media. If contrast dose is reduced, this leads to reduced kidney injuries, especially in oncologic patients who undergo several follow-up imaging examinations with contrast media administration. 

Recent studies have indicated that is possible to obtain a reduction from 50% to 70% in an iodinated contrast with adequate image quality using DECT with monoenergetic reconstructions at lower energy levels (55 keV) for the imaging of the aorta [99,100].

### 4.3. Reduce Metal Artifacts

One of the limitations of conventional CT is the excessive beam attenuation of metal implants, which leads to photon starvation, radiation scatter, the beam hardening artifact, excessive quantum noise, and scatter edge effects [101]. DECT can reduced beam hardening because monochromatic images at high keV are created from projection space data, which demonstrate the lower susceptibility this artifact [101,102].

**Figure 8:**
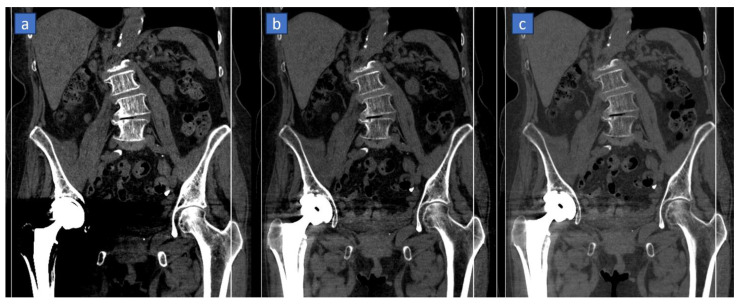
Reduction of metal-induced artifact in the pelvis of a 74-year-old woman suffering from rectal cancer. On the standard coronal CT reconstructed image, (**a**) heavy artifacts can be recognized around the total right hip prosthesis. The artifacts are well controlled on the monoenergetic reconstructed images obtained from DECT at 155 KeV (**b**) and above 170 KeV values (**c**), allowing for the correct visualization of pelvic structures.

In oncologic patients, this is useful for reducing streak artifacts due to the presence of metallic dental implants or dental amalgams that can cause image degradation in CT in the evaluation of salivary glands, tumors of the head and neck, or vertebral metastasis in dorsal or lumbar spine stabilization or in the pelvic area due to coxofemoral prostheses (Figure 8) [103,104]. Due to the aging of the general population and the increasing incidence of various tumors and metastases that progressively rise with age, it is now very common to evaluate oncology patients with metallic implant systems, stimulators, pacemakers, etc. In these cases, dual-energy CT imaging allows for optimized and tailored reconstructions to reduce artifacts and better visualize the structures of interest. The areas that can benefit the most from this application are the pelvis, the head–neck region, and the peri-prosthetic bones. The strength of the application lies in the ability to choose, in real-time, the best KeV level that reduces peri-prosthetic artifacts while maintaining reliable imaging appearances and densitometric values (Figure 5).

The same reconstructions could allow for the better evaluation of enhancing tumors with intracranial extent via the skull base foramina because of the reduction in artifacts originating from the bony skull [105].

CT artifacts from port-systems are a common problem in staging and restaging examinations and reduce image quality and diagnostic assessment in oncologic patients. High keV MEI+ enabled a significant reduction in artifacts from port-systems around the chamber and the catheter, leading to improved assessment of surrounding soft tissue [106].

A novel VMI reconstruction (S-MAR) also proved to be a promising method by which to reduce coil metal artifacts in cerebral CTA after coiled aneurysms and to elevate the vessel visualization adjacent to coils [107].

## 5. Bone Marrow Edema

Another useful application of DECT is the evaluation of bone marrow edema (BME) through the three-material decomposition of virtual non-calcium (VNCa) reconstructions. The calcium is calculated on dual-energy CT in the structures that can contain mineral material or calcifications which are subtracted from images to visualize the VNCa reconstructions, resulting in color-coded visualization of bone marrow edema [89]. Bone marrow pathologies are usually associated with a reduction in the fat component in the trabecular bone, replaced by water, hemorrhage, or cancer tissue depending on the underlying pathology [108].

Recently, many studies have assessed the high diagnostic accuracy in traumatic and non-traumatic BME in many skeletal segments, with excellent diagnostic performance for the spine (sensitivity: 0.84; specificity: 0.98) and appendicular skeleton (sensitivity: 0.84; specificity: 0.93) [109,110,111,112,113,114,115].

The spine represents the most common site of bone metastases, and most of the vertebral metastases are from breast, prostate, melanoma, renal carcinoma, and lung cancers. A contrast-enhanced CT scan is regularly performed in oncologic patients; however, the detection of bone marrow lesions on standard CT remains difficult. 

Color-coded VNCa reconstructions showed an high accuracy for qualitative assessment of metastatic lesions [116]. In particular, the use of low- and medium-calcium suppressions resulted in an increase of about 85% concerning sensitivity compared to conventional CT [117]. 

Moreover, DECT can depict BME associated with malignant lesions and help differentiate it from the linear pattern of BME associated with a fracture [116].

High diagnostic accuracy of VNCa reconstructions has also been demonstrated for assessing infiltrative oncologic diseases of vertebral bone marrow such as multiple myeloma, with high accuracy (ranging between 93% and 99%) [118].

Additionally, DECT can be leveraged opportunistically to assess bone density and muscle trophism, which often exhibit declines in oncology patients [119,120]. 

## 6. Lung Analysis

### 6.1. Pulmonary Thromboembolism

A further application of DECT lies in its potential to improve the visualization of pulmonary vasculature. It is particularly useful in the identification of pulmonary embolism since oncologic patients are more predisposed to develop this type of occlusive phenomena. 

Pulmonary embolism is often an incidental finding in those patients, and the phases of the examinations are focused on the venous phase. For this reason, a single venous phase reconstructed with low-keV images is suitable for an accurate diagnosis of pulmonary embolism in oncologic patients, with the added potential benefit of avoiding extra acquisitions and extra contrast media administration and limiting radiation exposure [88]. Virtual MEI+ images from the venous phase also showed higher contrast attenuation in more distal arteries, introducing the possibility of detecting smaller thrombi [121].

### 6.2. Lung Volumes and Perfusion

Secondly, it is also useful for the evaluation of pulmonary vasculature distribution for preoperative planning, together with the evaluation of lung volumes [122].

In fact, candidates for lung resection could benefit from the preoperative CT evaluation of lung volumes and assess the amount of residual parenchyma post-lobectomy or pneumectomy [123]. To do this, there are specific applications for DECT that allow for the calculation of total volumes, volumes compromised by disease, and volumes of healthy parenchyma, free from pathology. Moreover, dual-energy perfusion CT has been demonstrated to be more accurate than perfusion scintigraphy in predicting post-operative lung function [124].

It is also possible to quantify lung function in patients treated with radiation therapy for lung cancer and to assess the dosimetric impact of its integration in radiation therapy planning [125].

This method can be of great help to surgeons and radiotherapists for better planning and patient management. 

## 7. Conclusions

In conclusion, the applications described in this paper have a fundamental role in tumor assessment, and in oncologic imaging in general, for the diagnosis of primary tumors, local and metastatic staging, post-therapy evaluation, and the management of complications. In the future, the potential introduction of contrast agents with k-edges different from those of iodine could lead to a variety of additional applications and further developments.

## Data Availability

A data source can be provided upon reasonable request.

## References

[1] Wong A.J.N., Wong M., Kutschera P., Lau K.K. (2021). Dual-Energy CT in Musculoskeletal Trauma. Clin. Radiol..

[2] Rajiah P., Sundaram M., Subhas N. (2019). Dual-Energy CT in Musculoskeletal Imaging: What Is the Role Beyond Gout?. Am. J. Roentgenol..

[3] Cheraya G., Sharma S., Chhabra A. (2022). Dual Energy CT in Musculoskeletal Applications beyond Crystal Imaging: Bone Marrow Maps and Metal Artifact Reduction. Skelet. Radiol..

[4] Sandhu R., Aslan M., Obuchowski N., Primak A., Karim W., Subhas N. (2021). Dual-Energy CT Arthrography: A Feasibility Study. Skelet. Radiol..

[5] Lutz A.M. (2021). Using Dual-Energy CT for Painful Hip Arthroplasties. Radiology.

[6] Cicero G., Ascenti G., Albrecht M.H., Blandino A., Cavallaro M., D’Angelo T., Carerj M.L., Vogl T.J., Mazziotti S. (2020). Extra-Abdominal Dual-Energy CT Applications: A Comprehensive Overview. Radiol. Medica.

[7] Vlahos I., Jacobsen M.C., Godoy M.C., Stefanidis K., Layman R.R. (2022). Dual-Energy CT in Pulmonary Vascular Disease. Br. J. Radiol..

[8] Trabzonlu T.A., Mozaffary A., Kim D., Yaghmai V. (2020). Dual-Energy CT Evaluation of Gastrointestinal Bleeding. Abdom. Radiol..

[9] Tarkowski P., Czekajska-Chehab E. (2021). Dual-Energy Heart CT: Beyond Better Angiography—Review. J. Clin. Med..

[10] Dell’Aversana S., Ascione R., De Giorgi M., De Lucia D.R., Cuocolo R., Boccalatte M., Sibilio G., Napolitano G., Muscogiuri G., Sironi S. (2022). Dual-Energy CT of the Heart: A Review. J. Imaging.

[11] Agrawal M.D., Pinho D.F., Kulkarni N.M., Hahn P.F., Guimaraes A.R., Sahani D.V. (2014). Oncologic Applications of Dual-Energy CT in the Abdomen. RadioGraphics.

[12] Postma A.A., Das M., Stadler A.A.R., Wildberger J.E. (2015). Dual-Energy CT: What the Neuroradiologist Should Know. Curr. Radiol. Rep..

[13] Grajo J.R., Sahani D.V. (2018). Dual-Energy CT of the Abdomen and Pelvis: Radiation Dose Considerations. J. Am. Coll. Radiol..

[14] Danad I., Fayad Z.A., Willemink M.J., Min J.K. (2015). New Applications of Cardiac Computed Tomography. JACC Cardiovasc. Imaging.

[15] Carrascosa P., Deviggiano A., Rodriguez-Granillo G.A. (2017). Dual Energy Cardiac Computed Tomography. Minerva Cardiol. Angiol..

[16] Li W., Yu F., Liu M., Yan C. (2022). Clinical Value of Resting Cardiac Dual-Energy CT in Patients Suspected of Coronary Artery Disease. BMC Med. Imaging.

[17] Bodanapally U.K., Shanmuganathan K., Ramaswamy M., Tsymbalyuk S., Aarabi B., Parikh G.Y., Schwartzbauer G., Dreizin D., Simard M., Ptak T. (2019). Iodine-Based Dual-Energy CT of Traumatic Hemorrhagic Contusions: Relationship to In-Hospital Mortality and Short-Term Outcome. Radiology.

[18] Nair J.R., Burrows C., Jerome S., Ribeiro L., Larrazabal R., Gupta R., Yu E. (2020). Dual Energy CT: A Step Ahead in Brain and Spine Imaging. Br. J. Radiol..

[19] Kazimierczak W., Kazimierczak N., Serafin Z. (2023). Review of Clinical Applications of Dual-Energy CT in Patients after Endovascular Aortic Repair. J. Clin. Med..

[20] De Cecco C.N., Darnell A., Rengo M., Muscogiuri G., Bellini D., Ayuso C., Laghi A. (2012). Dual-Energy CT: Oncologic Applications. AJR Am. J. Roentgenol..

[21] Guerrini S., Bagnacci G., Perrella A., Meglio N.D., Sica C., Mazzei M.A. (2023). Dual Energy CT in Oncology: Benefits for Both Patients and Radiologists From an Emerging Quantitative and Functional Diagnostic Technique. Semin. Ultrasound CT MR.

[22] Ersahin D., Rasla J., Singh A. (2022). Dual Energy CT Applications in Oncological Imaging. Semin. Ultrasound CT MRI.

[23] Winkelmann M.T., Gassenmaier S., Walter S.S., Artzner C., Lades F., Faby S., Nikolaou K., Bongers M.N. (2022). Differentiation of Adrenal Adenomas from Adrenal Metastases in Single-Phased Staging Dual-Energy CT and Radiomics. Diagn. Interv. Radiol. Ank. Turk..

[24] Agostini A., Mari A., Lanza C., Schicchi N., Borgheresi A., Maggi S., Giovagnoni A. (2019). Trends in Radiation Dose and Image Quality for Pediatric Patients with a Multidetector CT and a Third-Generation Dual-Source Dual-Energy CT. Radiol. Medica.

[25] McCoombe K., Dobeli K., Meikle S., Llewellyn S., Kench P. (2022). Sensitivity of Virtual Non-Contrast Dual-Energy CT Urogram for Detection of Urinary Calculi: A Systematic Review and Meta-Analysis. Eur. Radiol..

[26] Connolly M.J., McInnes M.D.F., El-Khodary M., McGrath T.A., Schieda N. (2017). Diagnostic Accuracy of Virtual Non-Contrast Enhanced Dual-Energy CT for Diagnosis of Adrenal Adenoma: A Systematic Review and Meta-Analysis. Eur. Radiol..

[27] Takane Y., Sato K., Kageyama R., Takano H., Kayano S. (2022). Accuracy of Virtual Non-Contrast Images with Different Algorithms in Dual-Energy Computed Tomography. Radiol. Phys. Technol..

[28] Si-Mohamed S., Dupuis N., Tatard-Leitman V., Rotzinger D., Boccalini S., Dion M., Vlassenbroek A., Coulon P., Yagil Y., Shapira N. (2019). Virtual versus True Non-Contrast Dual-Energy CT Imaging for the Diagnosis of Aortic Intramural Hematoma. Eur. Radiol..

[29] Zhang P.P., Choi H.H., Ohliger M.A. (2022). Detection of Fatty Liver Using Virtual Non-Contrast Dual-Energy CT. Abdom. Radiol. N. Y..

[30] Mingkwansook V., Puwametwongsa K., Watcharakorn A., Dechasasawat T. (2022). Comparative Study of True and Virtual Non-Contrast Imaging Generated from Dual-Layer Spectral CT in Patients with Upper Aerodigestive Tract Cancer. Pol. J. Radiol..

[31] Wei X., Cao R., Li H., Long M., Sun P., Zheng Y., Li L., Yin J. (2022). Dual-Energy CT Iodine Map in Predicting the Efficacy of Neoadjuvant Chemotherapy for Hypopharyngeal Carcinoma: A Preliminary Study. Sci. Rep..

[32] Xu X.-Q., Zhou Y., Su G.-Y., Tao X.-W., Ge Y.-Q., Si Y., Shen M.-P., Wu F.-Y. (2022). Iodine Maps from Dual-Energy CT to Predict Extrathyroidal Extension and Recurrence in Papillary Thyroid Cancer Based on a Radiomics Approach. AJNR Am. J. Neuroradiol..

[33] Sun H., Hou X.-Y., Xue H.-D., Li X.-G., Jin Z.-Y., Qian J.-M., Yu J.-C., Zhu H.-D. (2015). Dual-Source Dual-Energy CT Angiography with Virtual Non-Enhanced Images and Iodine Map for Active Gastrointestinal Bleeding: Image Quality, Radiation Dose and Diagnostic Performance. Eur. J. Radiol..

[34] Li J.-X., Xie F.-J., Chen C.-H., Chen K.-M., Tsai C.-J. (2022). Dual-Energy Computed Tomography for Evaluation of Breast Cancer Follow-Ups: Comparison of Virtual Monoenergetic Images and Iodine-Map. Diagnostics.

[35] Mahmoudi S., Bernatz S., Althoff F.C., Koch V., Grünewald L.D., Scholtz J.-E., Walter D., Zeuzem S., Wild P.J., Vogl T.J. (2022). Dual-Energy CT Based Material Decomposition to Differentiate Intrahepatic Cholangiocarcinoma from Hepatocellular Carcinoma. Eur. J. Radiol..

[36] Lewin M., Laurent-Bellue A., Desterke C., Radu A., Feghali J.A., Farah J., Agostini H., Nault J.-C., Vibert E., Guettier C. (2022). Evaluation of Perfusion CT and Dual-Energy CT for Predicting Microvascular Invasion of Hepatocellular Carcinoma. Abdom. Radiol. N. Y..

[37] Liang H., Du S., Yan G., Zhou Y., Yang T., Zhang Z., Luo C., Liao H., Li Y. (2023). Dual-Energy CT of the Pancreas: Comparison between Virtual Non-Contrast Images and True Non-Contrast Images in the Detection of Pancreatic Lesion. Abdom. Radiol. N. Y..

[38] Frellesen C., Fessler F., Hardie A.D., Wichmann J.L., De Cecco C.N., Schoepf U.J., Kerl J.M., Schulz B., Hammerstingl R., Vogl T.J. (2015). Dual-Energy CT of the Pancreas: Improved Carcinoma-to-Pancreas Contrast with a Noise-Optimized Monoenergetic Reconstruction Algorithm. Eur. J. Radiol..

[39] Elsherif S.B., Zheng S., Ganeshan D., Iyer R., Wei W., Bhosale P.R. (2020). Does Dual-Energy CT Differentiate Benign and Malignant Ovarian Tumours?. Clin. Radiol..

[40] Daoud T., Morani A.C., Waters R., Bhosale P., Virarkar M.K. (2023). Diagnostic Approaches to Neuroendocrine Neoplasms of Unknown Primary Site. J. Comput. Assist. Tomogr..

[41] Lenga L., Lange M., Arendt C.T., Yel I., Booz C., Durden J., Leithner D., Vogl T.J., Albrecht M.H., Martin S.S. (2021). Can Dual-Energy CT-Based Virtual Monoenergetic Imaging Improve the Assessment of Hypodense Liver Metastases in Patients with Hepatic Steatosis?. Acad. Radiol..

[42] Wang N., Ju Y., Wu J., Liu A., Chen A., Liu J., Liu Y., Li J. (2019). Differentiation of Liver Abscess from Liver Metastasis Using Dual-Energy Spectral CT Quantitative Parameters. Eur. J. Radiol..

[43] Lenga L., Czwikla R., Wichmann J.L., Leithner D., Albrecht M.H., Booz C., Arendt C.T., Yel I., D’Angelo T., Vogl T.J. (2018). Dual-Energy CT in Patients with Colorectal Cancer: Improved Assessment of Hypoattenuating Liver Metastases Using Noise-Optimized Virtual Monoenergetic Imaging. Eur. J. Radiol..

[44] Si K., Wu H., Yang M., Guo Y., Zhang X., Ding C., Xue J., Han P., Li X. (2023). Utility of Dark-Blood Dual-Energy CT Images for Predicting Vascular Involvement and R0 Resection in Patients with Pancreatic Cancer. AJR Am. J. Roentgenol..

[45] Albrecht M.H., Vogl T.J., Martin S.S., Nance J.W., Duguay T.M., Wichmann J.L., De Cecco C.N., Varga-Szemes A., van Assen M., Tesche C. (2019). Review of Clinical Applications for Virtual Monoenergetic Dual-Energy CT. Radiology.

[46] Liang H., Zhou Y., Zheng Q., Yan G., Liao H., Du S., Zhang X., Lv F., Zhang Z., Li Y.-M. (2022). Dual-Energy CT with Virtual Monoenergetic Images and Iodine Maps Improves Tumor Conspicuity in Patients with Pancreatic Ductal Adenocarcinoma. Insights Imaging.

[47] Foti G., Fighera A., Campacci A., Natali S., Guerriero M., Zorzi C., Carbognin G. (2021). Diagnostic Performance of Dual-Energy CT for Detecting Painful Hip Prosthesis Loosening. Radiology.

[48] Darras K.E., Clark S.J., Kang H., Mohammed M.F., Barrett S., Chang S.D., Harris A.C., Nicolaou S., McLaughlin P.D. (2019). Virtual Monoenergetic Reconstruction of Contrast-Enhanced CT Scans of the Abdomen and Pelvis at 40 keV Improves the Detection of Peritoneal Metastatic Deposits. Abdom. Radiol. N. Y..

[49] Pourvaziri A., Parakh A., Cao J., Locascio J., Eisner B., Sahani D., Kambadakone A. (2022). Comparison of Four Dual-Energy CT Scanner Technologies for Determining Renal Stone Composition: A Phantom Approach. Radiology.

[50] McCollough C.H., Rajiah P.S. (2023). Milestones in CT: Past, Present, and Future. Radiology.

[51] Borges A.P., Antunes C., Curvo-Semedo L. (2023). Pros and Cons of Dual-Energy CT Systems: “One Does Not Fit All”. Tomography.

[52] Obmann M.M., Punjabi G., Obmann V.C., Boll D.T., Heye T., Benz M.R., Yeh B.M. (2022). Dual-Energy CT of Acute Bowel Ischemia. Abdom. Radiol. N. Y..

[53] Baxa J., Vondráková A., Matoušková T., Růžičková O., Schmidt B., Flohr T., Sedlmair M., Ferda J. (2014). Dual-Phase Dual-Energy CT in Patients with Lung Cancer: Assessment of the Additional Value of Iodine Quantification in Lymph Node Therapy Response. Eur. Radiol..

[54] Mohammadinejad P., Kwapisz L., Fidler J.L., Sheedy S.P., Heiken J.P., Khandelwal A., Wells M.L., Froemming A.T., Hansel S.L., Lee Y.S. (2021). The Utility of a Dual-Phase, Dual-Energy CT Protocol in Patients Presenting with Overt Gastrointestinal Bleeding. Acta Radiol. Open.

[55] Ozaki K., Higuchi S., Kimura H., Gabata T. (2022). Liver Metastases: Correlation between Imaging Features and Pathomolecular Environments. RadioGraphics.

[56] Silva A.C., Evans J.M., McCullough A.E., Jatoi M.A., Vargas H.E., Hara A.K. (2009). MR Imaging of Hypervascular Liver Masses: A Review of Current Techniques. Radiographics.

[57] Mastrodicasa D., Willemink M.J., Madhuripan N., Chima R.S., Ho A.A., Ding Y., Marin D., Patel B.N. (2021). Diagnostic Performance of Single-Phase Dual-Energy CT to Differentiate Vascular and Nonvascular Incidental Renal Lesions on Portal Venous Phase: Comparison with CT. Eur. Radiol..

[58] Hering D.A., Kröger K., Bauer R.W., Eich H.T., Haverkamp U. (2020). Comparison of Virtual Non-Contrast Dual-Energy CT and a True Non-Contrast CT for Contouring in Radiotherapy of 3D Printed Lung Tumour Models in Motion: A Phantom Study. Br. J. Radiol..

[59] Cao J., Lennartz S., Pisuchpen N., Mroueh N., Kongboonvijit S., Parakh A., Sahani D.V., Kambadakone A. (2022). Renal Lesion Characterization by Dual-Layer Dual-Energy CT: Comparison of Virtual and True Unenhanced Images. AJR Am. J. Roentgenol..

[60] Çamlıdağ İ., Nural M.S., Danacı M., Özden E. (2019). Usefulness of Rapid kV-Switching Dual Energy CT in Renal Tumor Characterization. Abdom. Radiol. N. Y..

[61] Foti G., Malleo G., Faccioli N., Guerriero A., Furlani L., Carbognin G. (2018). Characterization of Adrenal Lesions Using MDCT Wash-out Parameters: Diagnostic Accuracy of Several Combinations of Intermediate and Delayed Phases. Radiol. Medica.

[62] Nagayama Y., Inoue T., Oda S., Tanoue S., Nakaura T., Ikeda O., Yamashita Y. (2020). Adrenal Adenomas versus Metastases: Diagnostic Performance of Dual-Energy Spectral CT Virtual Noncontrast Imaging and Iodine Maps. Radiology.

[63] Badawy M., Gaballah A.H., Ganeshan D., Abdelalziz A., Remer E.M., Alsabbagh M., Westphalen A., Siddiqui M.A., Taffel M.T., Itani M. (2021). Adrenal Hemorrhage and Hemorrhagic Masses; Diagnostic Workup and Imaging Findings. Br. J. Radiol..

[64] Nelles C., Laukamp K.R., Große Hokamp N., Zaeske C., Celik E., Schoenfeld M.H., Borggrefe J., Kabbasch C., Schlamann M., Lennartz S. (2022). Virtual Non-Contrast Reconstructions Improve Differentiation between Vascular Enhancement and Calcifications in Stereotactic Planning CT Scans of Cystic Intracranial Tumors. Eur. J. Radiol..

[65] Lehti L., Söderberg M., Höglund P., Wassélius J. (2019). Comparing Arterial- and Venous-Phase Acquisition for Optimization of Virtual Noncontrast Images From Dual-Energy Computed Tomography Angiography. J. Comput. Assist. Tomogr..

[66] Lehti L., Söderberg M., Höglund P., Nyman U., Gottsäter A., Wassélius J. (2018). Reliability of Virtual Non-Contrast Computed Tomography Angiography: Comparing It with the Real Deal. Acta Radiol. Open.

[67] Zhang D., Wang X., Xue H., Jin Z., Sun H., Chen Y., He Y. (2016). Determinants of Detection of Stones and Calcifications in the Hepatobiliary System on Virtual Nonenhanced Dual-Energy CT. Chin. Med. Sci. J..

[68] Kim J.E., Lee J.M., Baek J.H., Han J.K., Choi B.I. (2012). Initial Assessment of Dual-Energy CT in Patients with Gallstones or Bile Duct Stones: Can Virtual Nonenhanced Images Replace True Nonenhanced Images?. AJR Am. J. Roentgenol..

[69] Lee M.H., Park H.J., Kim J.N., Kim M.S., Hong S.W., Park J.H., Kang C.H. (2022). Virtual Non-Contrast Images from Dual-Energy CT Angiography of the Abdominal Aorta and Femoral Arteries: Comparison with True Non-Contrast CT Images. Br. J. Radiol..

[70] Graser A., Johnson T.R.C., Hecht E.M., Becker C.R., Leidecker C., Staehler M., Stief C.G., Hildebrandt H., Godoy M.C.B., Finn M.E. (2009). Dual-Energy CT in Patients Suspected of Having Renal Masses: Can Virtual Nonenhanced Images Replace True Nonenhanced Images?. Radiology.

[71] Pulickal G.G., Singh D., Lohan R., Chawla A. (2019). Dual-Source Dual-Energy CT in Submandibular Sialolithiasis: Reliability and Radiation Burden. AJR Am. J. Roentgenol..

[72] Meyer M., Nelson R.C., Vernuccio F., González F., Farjat A.E., Patel B.N., Samei E., Henzler T., Schoenberg S.O., Marin D. (2019). Virtual Unenhanced Images at Dual-Energy CT: Influence on Renal Lesion Characterization. Radiology.

[73] Coursey C.A., Nelson R.C., Boll D.T., Paulson E.K., Ho L.M., Neville A.M., Marin D., Gupta R.T., Schindera S.T. (2010). Dual-Energy Multidetector CT: How Does It Work, What Can It Tell Us, and When Can We Use It in Abdominopelvic Imaging?. RadioGraphics.

[74] Yoon J.H., Lee J.M., Kim J.H., Lee K.-B., Kim H., Hong S.K., Yi N.-J., Lee K.-W., Suh K.-S. (2021). Hepatic Fibrosis Grading with Extracellular Volume Fraction from Iodine Mapping in Spectral Liver CT. Eur. J. Radiol..

[75] Li M., Fan Y., You H., Li C., Luo M., Zhou J., Li A., Zhang L., Yu X., Deng W. (2023). Dual-Energy CT Deep Learning Radiomics to Predict Macrotrabecular-Massive Hepatocellular Carcinoma. Radiology.

[76] Lee S.H., Lee J.M., Kim K.W., Klotz E., Kim S.H., Lee J.Y., Han J.K., Choi B.I. (2011). Dual-Energy Computed Tomography to Assess Tumor Response to Hepatic Radiofrequency Ablation: Potential Diagnostic Value of Virtual Noncontrast Images and Iodine Maps. Investig. Radiol..

[77] Lee J.-A., Jeong W.K., Kim Y., Song S.-Y., Kim J., Heo J.N., Park C.K. (2013). Dual-Energy CT to Detect Recurrent HCC after TACE: Initial Experience of Color-Coded Iodine CT Imaging. Eur. J. Radiol..

[78] Gupta S., Wagner-Bartak N., Jensen C.T., Hui A., Wei W., Lertdilok P., Qayyum A., Tamm E.P. (2016). Dual-Energy CT of Pancreatic Adenocarcinoma: Reproducibility of Primary Tumor Measurements and Assessment of Tumor Conspicuity and Margin Sharpness. Abdom. Radiol..

[79] Yuan J., Wang Y., Hu X., Shi S., Zhang N., Wang L., Deng W., Feng S.-T., Peng Z., Luo Y. (2023). Use of Dual-Layer Spectral Detector Computed Tomography in the Diagnosis of Pancreatic Neuroendocrine Neoplasms. Eur. J. Radiol..

[80] Lennartz S., Mager A., Große Hokamp N., Schäfer S., Zopfs D., Maintz D., Reinhardt H.C., Thomas R.K., Caldeira L., Persigehl T. (2021). Texture Analysis of Iodine Maps and Conventional Images for K-Nearest Neighbor Classification of Benign and Metastatic Lung Nodules. Cancer Imaging.

[81] Özdeniz İ., İdilman İ.S., Köklü S., Hamaloğlu E., Özmen M., Akata D., Karçaaltıncaba M. (2017). Dual-Energy CT Characteristics of Colon and Rectal Cancer Allows Differentiation from Stool by Dual-Source CT. Diagn. Interv. Radiol. Ank. Turk..

[82] May M.S., Wiesmueller M., Heiss R., Brand M., Bruegel J., Uder M., Wuest W. (2019). Comparison of Dual- and Single-Source Dual-Energy CT in Head and Neck Imaging. Eur. Radiol..

[83] Rizzo S., Radice D., Femia M., De Marco P., Origgi D., Preda L., Barberis M., Vigorito R., Mauri G., Mauro A. (2018). Metastatic and Non-Metastatic Lymph Nodes: Quantification and Different Distribution of Iodine Uptake Assessed by Dual-Energy CT. Eur. Radiol..

[84] Fervers P., Celik E., Bratke G., Maintz D., Baues C., Ruffing S., Pollman-Schweckhorst P., Kottlors J., Lennartz S., Große Hokamp N. (2021). Radiotherapy Response Assessment of Multiple Myeloma: A Dual-Energy CT Approach With Virtual Non-Calcium Images. Front. Oncol..

[85] Tsuchiya H., Tachibana Y., Kishimoto R., Omatsu T., Hotta E., Tanimoto K., Wakatsuki M., Obata T., Tsuji H. (2022). Dual-Energy Computed Tomography-Based Iodine Concentration Estimation for Evaluating Choroidal Malignant Melanoma Response to Treatment: Optimization and Primary Validation. Diagnostics.

[86] Hellbach K., Sterzik A., Sommer W., Karpitschka M., Hummel N., Casuscelli J., Ingrisch M., Schlemmer M., Graser A., Staehler M. (2017). Dual Energy CT Allows for Improved Characterization of Response to Antiangiogenic Treatment in Patients with Metastatic Renal Cell Cancer. Eur. Radiol..

[87] Jin K.N., De Cecco C.N., Caruso D., Tesche C., Spandorfer A., Varga-Szemes A., Schoepf U.J. (2016). Myocardial Perfusion Imaging with Dual Energy CT. Eur. J. Radiol..

[88] Foti G., Silva R., Faccioli N., Fighera A., Menghini R., Campagnola A., Carbognin G. (2021). Identification of Pulmonary Embolism: Diagnostic Accuracy of Venous-Phase Dual-Energy CT in Comparison to Pulmonary Arteries CT Angiography. Eur. Radiol..

[89] Parakh A., Lennartz S., An C., Rajiah P., Yeh B.M., Simeone F.J., Sahani D.V., Kambadakone A.R. (2021). Dual-Energy CT Images: Pearls and Pitfalls. Radiographics.

[90] Nagayama Y., Iyama A., Oda S., Taguchi N., Nakaura T., Utsunomiya D., Kikuchi Y., Yamashita Y. (2019). Dual-Layer Dual-Energy Computed Tomography for the Assessment of Hypovascular Hepatic Metastases: Impact of Closing k-Edge on Image Quality and Lesion Detectability. Eur. Radiol..

[91] De Cecco C.N., Caruso D., Schoepf U.J., De Santis D., Muscogiuri G., Albrecht M.H., Meinel F.G., Wichmann J.L., Burchett P.F., Varga-Szemes A. (2018). A Noise-Optimized Virtual Monoenergetic Reconstruction Algorithm Improves the Diagnostic Accuracy of Late Hepatic Arterial Phase Dual-Energy CT for the Detection of Hypervascular Liver Lesions. Eur. Radiol..

[92] Bhosale P., Le O., Balachandran A., Fox P., Paulson E., Tamm E. (2015). Quantitative and Qualitative Comparison of Single-Source Dual-Energy Computed Tomography and 120-kVp Computed Tomography for the Assessment of Pancreatic Ductal Adenocarcinoma. J. Comput. Assist. Tomogr..

[93] Marin D., Nelson R.C., Barnhart H., Schindera S.T., Ho L.M., Jaffe T.A., Yoshizumi T.T., Youngblood R., Samei E. (2010). Detection of Pancreatic Tumors, Image Quality, and Radiation Dose during the Pancreatic Parenchymal Phase: Effect of a Low-Tube-Voltage, High-Tube-Current CT Technique-Preliminary Results. Radiology.

[94] Lin X.Z., Wu Z.Y., Tao R., Guo Y., Li J.Y., Zhang J., Chen K.M. (2012). Dual Energy Spectral CT Imaging of Insulinoma—Value in Preoperative Diagnosis Compared with Conventional Multi-Detector CT. Eur. J. Radiol..

[95] Benveniste A.P., de Castro Faria S., Broering G., Ganeshan D.M., Tamm E.P., Iyer R.B., Bhosale P. (2017). Potential Application of Dual-Energy CT in Gynecologic Cancer: Initial Experience. AJR Am. J. Roentgenol..

[96] Patel B.N., Farjat A., Schabel C., Duvnjak P., Mileto A., Ramirez-Giraldo J.C., Marin D. (2018). Energy-Specific Optimization of Attenuation Thresholds for Low-Energy Virtual Monoenergetic Images in Renal Lesion Evaluation. AJR Am. J. Roentgenol..

[97] Shi C., Zhang H., Yan J., Wang B., Du L., Pan Z., Yan F. (2017). Decreased Stage Migration Rate of Early Gastric Cancer with a New Reconstruction Algorithm Using Dual-Energy CT Images: A Preliminary Study. Eur. Radiol..

[98] Li L., Zhao Y., Luo D., Yang L., Hu L., Zhao X., Wang Y., Liu W. (2018). Diagnostic Value of Single-Source Dual-Energy Spectral Computed Tomography in Differentiating Parotid Gland Tumors: Initial Results. Quant. Imaging Med. Surg..

[99] Johansen C.B., Martinsen A.C.T., Enden T.R., Svanteson M. (2022). The Potential of Iodinated Contrast Reduction in Dual-Energy CT Thoracic Angiography; an Evaluation of Image Quality. Radiography.

[100] Shuman W.P., O’Malley R.B., Busey J.M., Ramos M.M., Koprowicz K.M. (2017). Prospective Comparison of Dual-Energy CT Aortography Using 70% Reduced Iodine Dose versus Single-Energy CT Aortography Using Standard Iodine Dose in the Same Patient. Abdom. Radiol. N. Y..

[101] Nicolaou S., Liang T., Murphy D.T., Korzan J.R., Ouellette H., Munk P. (2012). Dual-Energy CT: A Promising New Technique for Assessment of the Musculoskeletal System. AJR Am. J. Roentgenol..

[102] Mallinson P.I., Coupal T.M., McLaughlin P.D., Nicolaou S., Munk P.L., Ouellette H.A. (2016). Dual-Energy CT for the Musculoskeletal System. Radiology.

[103] Chawla A., Srinivasan S., Lim T.-C., Pulickal G.G., Shenoy J., Peh W.C.G. (2017). Dual-Energy CT Applications in Salivary Gland Lesions. Br. J. Radiol..

[104] Sugawara H., Takayanagi T., Ishikawa T., Katada Y., Fukui R., Yamamoto Y., Suzuki S. (2020). New Fast kVp Switching Dual-Energy CT: Reduced Severity of Beam Hardening Artifacts and Improved Image Quality in Reduced-Iodine Virtual Monochromatic Imaging. Acad. Radiol..

[105] Gentili F., Guerrini S., Mazzei F.G., Monteleone I., Di Meglio N., Sansotta L., Perrella A., Puglisi S., De Filippo M., Gennaro P. (2020). Dual Energy CT in Gland Tumors: A Comprehensive Narrative Review and Differential Diagnosis. Gland Surg..

[106] Laukamp K.R., Zopfs D., Wagner A., Lennartz S., Pennig L., Borggrefe J., Ramaiya N., Große Hokamp N. (2019). CT Artifacts from Port Systems: Virtual Monoenergetic Reconstructions from Spectral-Detector CT Reduce Artifacts and Improve Depiction of Surrounding Tissue. Eur. J. Radiol..

[107] Zheng H., Yang M., Jia Y., Zhang L., Sun X., Zhang Y., Nie Z., Wu H., Zhang X., Lei Z. (2022). A Novel Subtraction Method to Reduce Metal Artifacts of Cerebral Aneurysm Embolism Coils. Clin. Neuroradiol..

[108] Steinmetz M.P., Mekhail A., Benzel E.C. (2001). Management of Metastatic Tumors of the Spine: Strategies and Operative Indications. Neurosurg. Focus.

[109] Pache G., Krauss B., Strohm P., Saueressig U., Blanke P., Bulla S., Schäfer O., Helwig P., Kotter E., Langer M. (2010). Dual-Energy CT Virtual Noncalcium Technique: Detecting Posttraumatic Bone Marrow Lesions—Feasibility Study. Radiology.

[110] Foti G., Guerriero M., Faccioli N., Fighera A., Romano L., Zorzi C., Carbognin G. (2021). Identification of Bone Marrow Edema around the Ankle Joint in Non-Traumatic Patients: Diagnostic Accuracy of Dual-Energy Computed Tomography. Clin. Imaging.

[111] Foti G., Faccioli N., Silva R., Oliboni E., Zorzi C., Carbognin G. (2020). Bone Marrow Edema around the Hip in Non-Traumatic Pain: Dual-Energy CT vs MRI. Eur. Radiol..

[112] Foti G., Catania M., Caia S., Romano L., Beltramello A., Zorzi C., Carbognin G. (2019). Identification of Bone Marrow Edema of the Ankle: Diagnostic Accuracy of Dual-Energy CT in Comparison with MRI. Radiol. Medica.

[113] Foti G., Mantovani W., Faccioli N., Crivellari G., Romano L., Zorzi C., Carbognin G. (2021). Identification of Bone Marrow Edema of the Knee: Diagnostic Accuracy of Dual-Energy CT in Comparison with MRI. Radiol. Medica.

[114] Frellesen C., Azadegan M., Martin S.S., Otani K., DʼAngelo T., Booz C., Eichler K., Panahi B., Kaup M., Bauer R.W. (2018). Dual-Energy Computed Tomography-Based Display of Bone Marrow Edema in Incidental Vertebral Compression Fractures: Diagnostic Accuracy and Characterization in Oncological Patients Undergoing Routine Staging Computed Tomography. Investig. Radiol..

[115] Suh C.H., Yun S.J., Jin W., Lee S.H., Park S.Y., Ryu C.-W. (2018). Diagnostic Performance of Dual-Energy CT for the Detection of Bone Marrow Oedema: A Systematic Review and Meta-Analysis. Eur. Radiol..

[116] Gosangi B., Mandell J.C., Weaver M.J., Uyeda J.W., Smith S.E., Sodickson A.D., Khurana B. (2020). Bone Marrow Edema at Dual-Energy CT: A Game Changer in the Emergency Department. RadioGraphics.

[117] Abdullayev N., Große Hokamp N., Lennartz S., Holz J.A., Romman Z., Pahn G., Neuhaus V., Maintz D., Krug B., Borggrefe J. (2019). Improvements of Diagnostic Accuracy and Visualization of Vertebral Metastasis Using Multi-Level Virtual Non-Calcium Reconstructions from Dual-Layer Spectral Detector Computed Tomography. Eur. Radiol..

[118] Kosmala A., Weng A.M., Krauss B., Knop S., Bley T.A., Petritsch B. (2018). Dual-Energy CT of the Bone Marrow in Multiple Myeloma: Diagnostic Accuracy for Quantitative Differentiation of Infiltration Patterns. Eur. Radiol..

[119] Gruenewald L.D., Koch V., Martin S.S., Yel I., Mahmoudi S., Bernatz S., Eichler K., Gruber-Rouh T., Pinto Dos Santos D., D’Angelo T. (2023). Dual-Energy CT-Based Opportunistic Volumetric Bone Mineral Density Assessment of the Distal Radius. Radiology.

[120] Heinrich A., Schenkl S., Buckreus D., Güttler F.V., Teichgräber U.K.-M. (2022). CT-Based Thermometry with Virtual Monoenergetic Images by Dual-Energy of Fat, Muscle and Bone Using FBP, Iterative and Deep Learning-Based Reconstruction. Eur. Radiol..

[121] Patel A.A., Sutphin P.D., Xi Y., Abbara S., Kalva S.P. (2019). Arterial Phase CTA Replacement by a Virtual Arterial Phase Reconstruction from a Venous Phase CTA: Preliminary Results Using Detector-Based Spectral CT. Cardiovasc. Interv. Radiol..

[122] Choe J., Lee S.M., Chae E.J., Lee S.M., Kim Y.-H., Kim N., Seo J.B. (2017). Evaluation of Postoperative Lung Volume and Perfusion Changes by Dual-Energy Computed Tomography in Patients with Lung Cancer. Eur. J. Radiol..

[123] Suh Y.J., Lee C.Y., Lee S., Kim H.E., Kim Y.J. (2023). Patterns of Postoperative Changes in Lung Volume and Perfusion Assessed by Dual-Energy CT: Comparison of Lobectomy and Limited Resection. AJR Am. J. Roentgenol..

[124] Chae E.J., Kim N., Seo J.B., Park J.-Y., Song J.-W., Lee H.J., Hwang H.J., Lim C., Chang Y.J., Kim Y.H. (2013). Prediction of Postoperative Lung Function in Patients Undergoing Lung Resection: Dual-Energy Perfusion Computed Tomography versus Perfusion Scintigraphy. Investig. Radiol..

[125] Bahig H., Campeau M.-P., Lapointe A., Bedwani S., Roberge D., de Guise J., Blais D., Vu T., Lambert L., Chartrand-Lefebvre C. (2017). Phase 1-2 Study of Dual-Energy Computed Tomography for Assessment of Pulmonary Function in Radiation Therapy Planning. Int. J. Radiat. Oncol. Biol. Phys..

